# Early Anomaly Pre-Warning of Buried Pipelines via Dynamic Acceleration Signals: An ICEEMDAN-LSTM Framework

**DOI:** 10.3390/s26113463

**Published:** 2026-05-30

**Authors:** Ying-Qing Guo, Zhi-Xin Zhu, Zhi-Heng Xia, Xu-Lei Zang, Jin-Bao Li

**Affiliations:** 1 College of Mechanical and Electronic Engineering, Nanjing Forestry University, Nanjing 210037, China; zhixinzhu@njfu.edu.cn; 2 China–Pakistan Belt and Road Joint Laboratory on Smart Disaster Prevention of Major Infrastructures, Southeast University, Nanjing 210096, China; zhiheng_xia@163.com (Z.-H.X.); zxlseu@seu.edu.cn (X.-L.Z.); 3 Jiangsu Southeast Special Engineering & Technology Co., Ltd., Nanjing 210009, China; ljb1958@163.com

**Keywords:** buried natural gas pipelines, structural health monitoring (SHM), acceleration signal analysis, improved complete ensemble empirical mode decomposition with adaptive noise (ICEEMDAN), long short-term memory (LSTM)

## Abstract

**Highlights:**

1. An intelligent ICEEMDAN-LSTM framework is developed to achieve early-stage anomaly pre-warning for buried natural gas pipelines.
2. A unified data-driven model successfully integrates adaptive signal decomposition and sequential learning to enhance weak transient feature extraction from non-stationary vibrations.
3. A novel two-stage complementary validation paradigm is formulated, effectively bridging unsupervised data-driven field baselines with multi-physics fluid-structure interaction digital twins.
4. The framework delivers physically interpretable pre-warning capabilities, systematically overcoming the critical industry bottleneck of labeled failure data scarcity.

**What are the main findings?**

- A physically-informed health monitoring framework integrating ICEEMDAN decomposition and LSTM classification effectively extracts multi-scale features and isolates transient precursor anomalies from complex acceleration signals.
- A two-stage complementary validation, coupling continuous field construction logs with FSI digital twins, rigorously verifies the system’s sensitivity to genuine structural degradation.

**What are the implications of the main findings?**

- The proposed ICEEMDAN-LSTM framework provides a highly reliable, physically interpretable data-driven methodology for the intelligent structural health monitoring of buried pipelines, particularly in complex operating environments subject to heavy background noise and dynamic disturbances.
- By introducing a novel two-stage complementary validation strategy and leveraging unsupervised clustering, the “cold-start” problem—the complete lack of destructive labeled data—was successfully addressed, offering a scalable and practical solution for the early anomaly pre-warning of critical infrastructure.

**Abstract:**

Structural health monitoring of buried pipelines is essential due to their exposure to corrosion, impact loads, and geotechnical disturbances, which may induce abnormal vibration responses. Acceleration signals provide direct and sensitive measurements of buried pipeline structural dynamic behavior, and are therefore suitable for early anomaly identification. An acceleration-based intelligent framework integrating Improved Complete Ensemble Empirical Mode Decomposition with Adaptive Noise (ICEEMDAN) and a Long Short-Term Memory (LSTM) network is proposed for buried pipeline condition recognition. First, the raw acceleration signals are decomposed into a set of intrinsic mode functions (IMFs) using ICEEMDAN to enhance time–frequency resolution and isolate weak transient impact components associated with buried pipeline structural anomalies. Subsequently, multi-scale features extracted from the IMFs are fused and fed into an LSTM network to capture temporal dependencies and perform supervised health state classification. Experimental results demonstrate that the proposed framework achieves an F1-score of 0.70 and a Precision–Recall AUC of 0.72 for identifying anomalies. Furthermore, cross-validation utilizing multi-source field data (dynamic acceleration and quasi-static strain) confirms the model’s physical interpretability and its stable performance under severe noise interference. The results validate the feasibility of combining advanced signal decomposition with deep learning techniques for buried pipeline anomaly pre-warning, providing a rigorous methodological basis for the safe operation of critical energy infrastructures.

## 1. Introduction

As typical thin-walled structures, buried natural gas pipelines are highly susceptible to fatigue and structural failure due to complex geological environments, surface loads, and anthropogenic activities. This necessitates high-accuracy structural health monitoring (SHM) methods to ensure operational safety [1,2,3]. Vibration-based SHM using acceleration sensors is widely applied for nondestructive pipeline testing due to its ability to capture transient fault impacts with high precision and low noise [4,5,6,7,8,9]. To enhance automated fault extraction and diagnostic robustness under variable operating conditions, recent international studies have successfully integrated these acceleration signals with advanced deep learning models, such as CNNs and ANNs [10,11,12]. However, despite these advancements, conventional acceleration-based neural networks still face substantial challenges when processing real-world data, particularly regarding heavy environmental background noise, ambiguous time–frequency signatures, and poor generalizability across different pipeline topologies.

Operating in complex soil environments, buried pipelines generate highly non-stationary acceleration signals where critical transient anomalies, such as mechanical impacts or structural degradation, are frequently drowned out by dense environmental noise. This issue complicates feature extraction. While various time–frequency techniques exist—including Wavelet Decomposition [13], LMD [14], KPCA [15], and SSA [16]—Empirical Mode Decomposition (EMD) [17] is particularly suited for such signals. However, capturing early-stage pipeline damage requires extracting intermittent, low-amplitude structural shocks, which inevitably cause mode-mixing and spurious component issues in the original EMD. To resolve the mode-mixing issues inherent in EMD, joint diagnostic frameworks combining advanced ultrasonic guided waves with targeted wavelet denoising have been successfully formulated to enhance localized pipeline damage identification [18,19]. Noise-assisted variants like MI-EEMD [20] and CEEMDAN [21] have been progressively deployed to mitigate these effects, and they frequently introduce residual noise that obscures subtle pipeline pre-warning signatures. To resolve this logical bottleneck, the Improved CEEMDAN (ICEEMDAN) algorithm has emerged, demonstrating superior performance in adaptively decomposing acceleration signals into robust Intrinsic Mode Functions (IMFs) that accurately preserve multi-scale time-varying dynamic patterns [22].

Furthermore, pipeline health evolution exhibits strong temporal short- and long-term dependencies that static, instantaneous features fail to capture. Long Short-Term Memory (LSTM) networks excel in modeling these long-span temporal correlations for pipeline fault diagnosis [23,24,25,26]. Recent research has further expanded the robustness of LSTM-based frameworks in operational pipeline environments via optimization algorithms (e.g., PSO [27,28], DBO [29]) and hybrid architectures (e.g., CNN-LSTM [30], AM-LSTM [31]). Despite these successes, the diagnostic performance of the LSTM layer remains strictly dependent on the quality and resolution of the input feature space. Because conventional LSTMs lack intrinsic time–frequency resolution, training them directly on raw pipeline acceleration signals—which are heavily corrupted by complex soil–structure interactions—renders the network highly susceptible to overfitting and misclassification. Consequently, overcoming the LSTM’s vulnerability to input noise by constructing a high-fidelity preprocessing paradigm remains a critical engineering challenge in global pipeline SHM.

To systematically address these interconnected limitations, this paper proposes an acceleration-based ICEEMDAN-LSTM framework featuring a high-resolution time–frequency input space (WPE-SWT-t-SNE) to accurately capture weak, masked early pipeline degradation signatures. Crucially, a novel two-stage complementary validation strategy combining data-driven field baselines with multi-physics fluid–structure interaction (FSI) digital twins is formulated to systematically overcome the engineering bottleneck of labeled failure data scarcity. The main technical advantage of the proposed framework is its exceptional diagnostic fidelity and heightened sensitivity in capturing early-stage pipeline performance degradation without requiring extensive empirical parameter tuning, thereby ensuring seamless compatibility and deployment with existing industrial SCADA networks. Nevertheless, a recognized limitation of this study remains its current validation within controlled physical experimental loops and localized pipe–soil boundary configurations, which necessitates future extensive field verification on active, large-scale transmission lines with diverse geological variations.

## 2. Problem Formulation and Validation Strategy

Evaluating structural health monitoring (SHM) algorithms for operational urban buried gas pipelines entails a fundamental paradox: safety regulations strictly prohibit inducing physical damage, yet the Cold-start problem—the total absence of labeled failure data—remains a primary barrier to deploying supervised models.

**Figure 1:**
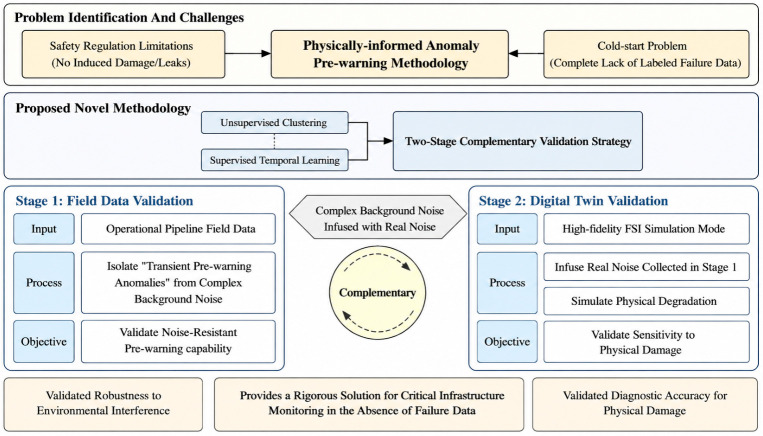
The framework of the proposed physically informed anomaly pre-warning methodology with a two-stage complementary validation strategy.

To overcome the Cold-start problem and the inherent absence of labeled failure data in operational buried pipelines, a physically informed anomaly pre-warning methodology is proposed. As illustrated in Figure 1, the framework introduces a novel Two-Stage Complementary Validation Strategy that systematically bridges the gap between unsupervised clustering (for baseline definition) and supervised temporal learning (for anomaly identification). This strategic integration explicitly translates statistical data fluctuations into actionable structural warnings, ensuring both algorithmic robustness and physical interpretability.

Stage 1: Field Data Evaluation for Noise-Resistant Pre-warning. Within this complementary architecture, Stage 1 establishes a statistical foundation by leveraging multi-sensor field data collected from operational, zero-failure environments. The unsupervised clustering layer maps the continuous, high-dimensional boundaries of the pipeline’s “healthy baseline” under highly variable ambient conditions. To achieve high noise resistance, the algorithm systematically cross-references steady-state operations against localized, non-destructive transient anomalies—such as adjacent heavy-traffic vibrations, soil settlements, and minor surface impacts. By quantifying the mathematical variance and spectral density of these environmental interferences, Stage 1 defines a clear rejection threshold for non-damage fluctuations, ensuring that the warning system possesses high robustness against false positives before mapping any structural degradation.

Stage 2: Digital Twin-Driven Physical Sensitivity Validation. To counter the “lack of anomaly information,” Stage 2 implements a high-fidelity Digital Twin framework powered by multi-physics Fluid–Structure Interaction (FSI) simulations. The virtual pipeline twin accurately models the coupled dynamic behavior of high-pressure natural gas transit and complex nonlinear soil constraints. To execute rigorous physical sensitivity validation, structural micro-degradations—specifically geometric dents of varying depths are parametrically introduced into the simulation domain. Crucially, the real-world ambient background noise matrices isolated during Stage 1 are synthetically superimposed onto these synthetic FSI acceleration responses. This bidirectional noise infusion allows the supervised deep sequence network to train on physical damage profiles that inherit authentic environmental attenuation properties. The digital twin is considered validated when the structural stress–strain thresholds and simulated modal frequencies demonstrate a strict mathematical alignment with the empirical metrics, successfully bridging the gap between abstract statistical deviations and explicit physical damage.

Consequently, this integrated validation architecture ensures a rigorous, dual-verification paradigm: while real-world field data explicitly evaluates the framework’s robustness against complex urban environmental interferences, the multi-physics digital twin comprehensively validates its diagnostic sensitivity to explicit structural degradation. Ultimately, the systematic fusion of data-driven anomaly detection and physical-domain simulations establishes a highly generalizable and closed-loop solution to the inherent scarcity of labeled failure data in critical infrastructure monitoring, providing both statistical reliability and physical interpretability.

## 3. Acceleration-Based ICEEMDAN-LSTM Framework for Buried Pipeline Structural Health Recognition

### 3.1. Multi-Dimensional Feature Space Construction via ICEEMDAN

In the analysis of buried pipeline vibration acceleration signals, the raw data are often subject to multi-source noise interference and exhibit pronounced non-stationary and nonlinear characteristics. Traditional methods, such as the Fourier Transform and Wavelet Decomposition, require predefined basis functions or assumptions of signal stationarity, making them inadequate for effectively capturing transient shocks and multi-scale features. To address this limitation, the Improved Complete Ensemble Empirical Mode Decomposition with Adaptive Noise (ICEEMDAN) method is employed to perform the multi-scale decomposition of vibration acceleration signals acquired from buried pipelines, thereby facilitating robust subsequent feature extraction.

Improved Complete Ensemble Empirical Mode Decomposition with Adaptive Noise (ICEEMDAN) improves decomposition stability and accuracy by introducing adaptive noise and a complete ensemble strategy. It is particularly well-suited for analyzing non-stationary and nonlinear signals, such as buried pipeline acceleration data [32]. To strictly eliminate the risk of data leakage, the ICEEMDAN algorithm is applied independently to each segmented 2 s measurement window (containing 100 data points) rather than the entire long-duration signal. This ensures that the construction of Intrinsic Mode Functions (IMFs) for any given sample relies solely on local extrema within that specific window, preventing the inclusion of ‘future’ information from subsequent time steps. This methodological rigor ensures that the training and testing phases are completely isolated, providing a scientifically valid foundation for evaluating the model’s performance.

First, the initial residual is defined as

(1) $$r_{0}\left(\tau \right)=x\left(\tau \right)$$

where τ denotes the time variable.

Subsequently, Additive white Gaussian noise is added to the residual $r_{0}\left(\tau \right)$:

(2) $$x^{\left(i\right)}\left(\tau \right)=x\left(\tau \right)+\beta_{0}E_{1}\left(w^{\left(i\right)}\left(\tau \right)\right)$$

where $w^{\left(i\right)}\left(\tau \right)$ is the i-th realization of Additive white Gaussian noise, $E_{1}\left(\cdot \right)$ denotes the operator of EMD that extracts the first IMF component in a single decomposition step, and $\beta_{0}$ is the noise weighting coefficient (typically 0.2–0.3).

The noise-added signal $x^{\left(i\right)}\left(\tau \right)$ is then decomposed by EMD to obtain the first intrinsic mode function (IMF_1_):

(3) $${IMF}_{1}=\frac{1}{N}\sum_{i=1}^{N}E_{1}\left(x^{\left(i\right)}\left(\tau \right)\right)$$

The residual is updated accordingly as

(4) $$r_{1}\left(\tau \right)=x\left(\tau \right)-{IMF}_{1}\left(\tau \right)$$

After eliminating the high-frequency noise, the procedure of noise injection and decomposition is iteratively applied to the updated residual $r_{k-1}\left(\tau \right)$, yielding the subsequent *K*-th IMF (IMF_k_) in the overall decomposition sequence:

(5) $${IMF}_{k}\left(\tau \right)=\frac{1}{N}\sum_{i=1}^{N}E_{1}\left(r_{k-1}\left(\tau \right)\right)+\beta_{k-1}E_{k}\left(w^{\left(i\right)}\left(\tau \right)\right)$$

When the residual becomes a monotonic function, the decomposition process terminates, and the final residual represents the trend of the signal. Through the ICEEMDAN procedure, the signal can be decomposed into a set of IMFs ranging from high to low frequency. Different frequency bands are associated with different anomaly types: IMF_1_–IMF_3_ capture high-frequency transient impacts and elastic wave propagation within the buried pipeline wall, often associated with local stress releases. IMF_4_–IMF_6_ reflect the global oscillatory modes of the soil-pipeline system, representing its macro-dynamic equilibrium. By performing feature extraction and post-processing on different IMFs, additional informative characteristics can be obtained. To enhance the discriminative capability of high-frequency components, stationary wavelet transforms (SWTs) are applied to selected IMFs for signal reconstruction and feature extraction, thereby providing supplementary spectral information.

Weighted permutation entropy (WPE) is systematically employed to quantify the nonlinear dynamic complexity and structural regularity shifts embedded within the pipeline’s vibration acceleration signals. In buried structural health monitoring, external anthropogenic disturbances or early-stage macro-structural variations generate non-stationary elastic stress waves that typically concentrate within specific, localized frequency bands. At the embryonic phase of pipeline degradation, these anomalous structural events manifest as transient, low-amplitude mechanical impacts, which subsequently accumulate and evolve into persistent structural deformation patterns. While classical permutation entropy (PE) maps phase-space reconstruction vectors into ordinal patterns based strictly on their temporal sequence order, it completely discards the amplitude information, rendering it insensitive to amplitude mutations under heavy background noise. WPE surmounts this critical limitation by incorporating an amplitude-weighting factor—derived from the variance or localized power of each reconstructed sub-sequence vector—into the probability distribution calculation of the permutation patterns. Consequently, when transient mechanical impacts from third-party excavation or heavy vehicular loading strike the pipe body, they abruptly alter the structural boundary state and disrupt the intrinsic ordinal regularity of the acceleration series. By capturing these subtle phase-space structural mutations, WPE exhibits heightened sensitivity to weak, masked pre-warning signatures where localized amplitude variations play a decisive role, effectively separating true pipe-wall micro-perturbations from dense ambient soil–structure interaction noise.

Based on the advanced adaptive decomposition paradigm, the ICEEMDAN algorithm systematically extracts multiple IMFs from the raw vibration series, effectively isolating the structural responses of the buried pipeline across distinct time–frequency scales. To fully exploit the latent health-related patterns embedded within these discrete non-stationary modes, a comprehensive joint multi-source feature space is constructed by calculating both the nonlinear complexity metrics and the multi-scale frequency-domain energy indicators across the representative IMF layers. Specifically, for the high-priority modes (IMF1–IMF6), WPE is computed to trace the sequential configuration complexity shifts, while the SWT is simultaneously applied to extract the detail coefficient energy distributions, capturing the exact spectral power redistribution induced by localized pipeline structural anomalies.

**Figure 2:**
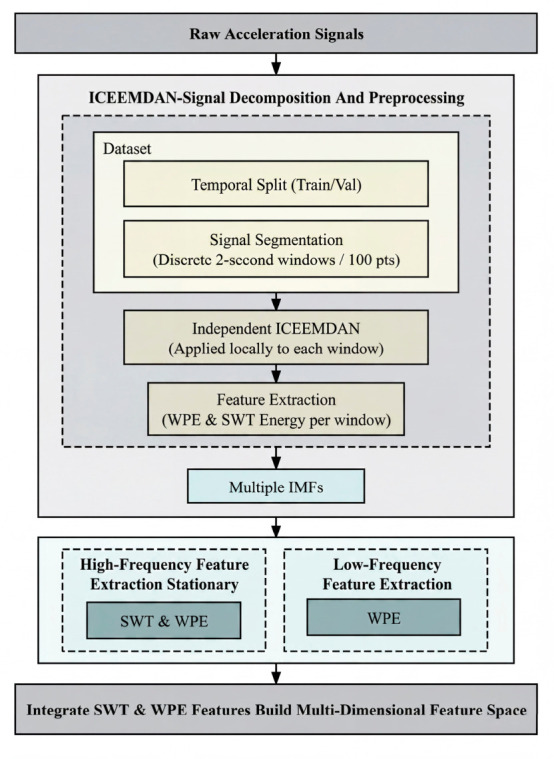
Construction of the multi-dimensional feature system.

For IMF_1_–IMF_6_, the WPE features combined with SWT-based features are concatenated to construct the comprehensive multi-dimensional feature vector, i.e., $F=\left[Feature\_0,Feature\_1,\ldots \right]$. The complete process is shown in Figure 2. By fusing these nonlinear complexity metrics with frequency-domain energy indicators, the resulting high-dimensional representation provides a robust basis for the subsequent isolation of subtle anomalies from dense background noise.

### 3.2. Temporal Sequence Modeling and Anomaly Identification via LSTM

Due to the strong temporal dependency and dynamic evolution inherent in acceleration signals, the discriminative information is not only contained in instantaneous feature values but also manifested in the evolving patterns across time. Consequently, conventional static classifiers, such as support vector machines (SVMs) and random forests, are insufficient for fully capturing the temporal patterns of vibration signals. To address this limitation, the LSTM network is integrated with the ICEEMDAN algorithm for the analysis of buried pipeline acceleration signals. Through this strategic combination of advanced signal decomposition and deep learning, the accuracy of anomaly identification is substantially enhanced.

Long Short-Term Memory (LSTM), as a variant of Recurrent Neural Networks (RNNs), addresses the issues of gradient vanishing and gradient explosion inherent in traditional RNNs [33]. The core architecture of an LSTM is the cell state, which regulates the flow and updating of internal information through the coordinated action of three gating mechanisms: the forget gate, input gate, and output gate.

Assume that at the time t, the input feature vector is $x_{t}$ (representing the previously discussed multi-sensor fusion features), the hidden state from the previous step is $h_{t-1}$, and the previous cell state is $C_{t-1}$. The LSTM unit updates and outputs information via the following procedure:

(1) Forget Phase (Forget Gate): The forget gate $f_{t}$ determines the proportion of information to be retained from the previous cell state $C_{t-1}$. By processing $h_{t-1}$ and $x_{t}$ through a Sigmoid activation function, the gate outputs a vector with values ranging from 0 to 1. The mathematical expression is:
  (6) $$f_{t}=\sigma \left(W_{f}\left[h_{t-1},x_{t}\right]+b_{f}\right)$$
(2) Input and Update Phase (Input Gate and Candidate Memory): This stage determines which new multi-sensor information should be integrated into the cell state. First, the input gate $i_{t}$ identifies the values to be updated; subsequently, a candidate cell state vector $\tilde{C_{t}}$ is constructed using a Tanh function. The corresponding formulas are:
  (7) $$i_{t}=\sigma \left(W_{i}\left[h_{t-1},x_{t}\right]+b_{i}\right)$$
  (8) $$\tilde{C_{t}}=tanh\left(W_{C}\left[h_{t-1},x_{t}\right]+b_{c}\right)$$
(3) Cell State Update: The cell state is updated by combining the outputs of the forget and input gates. The previous state $C_{t-1}$ is multiplied by $f_{t}$ to eliminate redundant historical data (e.g., environmental noise), while the element-wise product of $i_{t}$ and $\tilde{C_{t}}$ is added to incorporate salient current features (e.g., instantaneous impulses). The updated cell state $\tilde{C_{t}}$ is defined as:
  (9) $$C_{t}=f_{t}{\cdot C}_{t-1}+i_{t}\cdot \tilde{C_{t}}$$
(4) Output Phase (Output Gate): The final output is derived from the updated cell state. The output gate $o_{t}$ determines which portions of the cell state are exported. The cell state $C_{t}$ is normalized to the range [−1, 1] via a Tanh function and then multiplied by the output gate to produce the final hidden state $h_{t}$:
  (10) $$o_{t}=\sigma \left(W_{o}\left[h_{t-1},x_{t}\right]+b_{o}\right)$$
  (11) $$h_{t}=o_{t}\tanh\left(C_{t}\right)$$

To track the time-varying degradation trajectories of buried pipelines, an LSTM-based deep neural network was constructed. The architecture sequentially comprises an input layer, an LSTM hidden layer configured with 32 hidden units, and an output layer for loss computation. Because external physical interferences induce continuous structural vibration responses rather than isolated discrete points, the continuous field monitoring samples are segmented into 10 × 9 sequences via a 5-step overlapping sliding window mechanism. This temporal overlapping operation strictly ensures that the integrity of continuous transient physical shocks is preserved across data frames, preventing mechanical wave boundary truncation. Furthermore, this topological design yields a highly compact network comprising exactly 5504 trainable parameters. Such a lightweight configuration is explicitly optimized to meet the real-time processing and low-latency constraints of operational pipeline monitoring systems, avoiding the computational bottlenecks typical of over-parameterized deep learning models.

**Figure 3:**
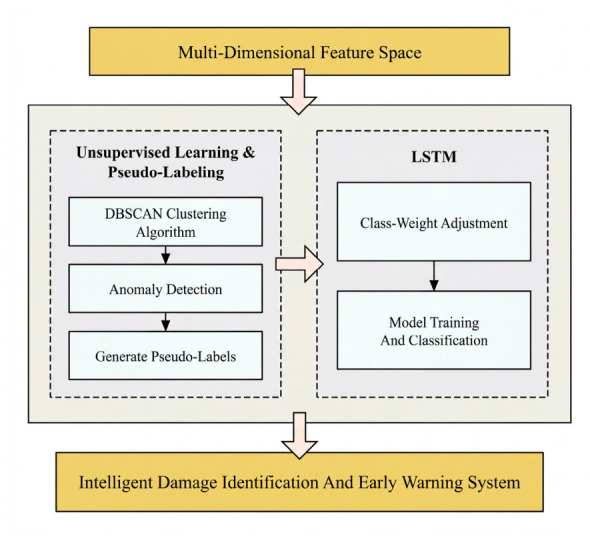
Anomaly identification and intelligent pre-warning.

Supervised temporal modeling via LSTM inherently demands comprehensive labeled datasets. However, in operational buried pipelines, the inability to fully control complex soil environments and the strict prohibition of inducing destructive physical failures result in a severe scarcity of labeled anomaly data. To bridge this gap, unsupervised learning mechanisms must be introduced to autonomously isolate anomaly patterns from vast amounts of unlabeled monitoring data, thereby generating reliable pseudo-labels prior to sequence modeling. Commonly used unsupervised methods include K-means [34] and DBSCAN [35], while typical dimensionality reduction techniques include principal component analysis (PCA) [36] and t-distributed stochastic neighbor embedding (t-SNE) [37]. The final deployment of specific unsupervised algorithms is strictly dictated by the mathematical topology and statistical distribution of the extracted multi-dimensional feature space, which will be detailed in Section 4. Guided by this data-driven rationale, an integrated intelligent pre-warning architecture is established to systematically resolve the engineering bottlenecks of zero-failure cold starts and extreme class imbalance. The overall technical roadmap of this framework is illustrated in Figure 3.

## 4. Field Data Analysis and Anomaly Pre-Warning Capacity

### 4.1. Data Source and Monitoring Setup

The experimental data were collected from an in situ monitoring system installed along a section of the West–East Gas Pipeline. The buried pipeline has an outer diameter of 1016 mm, a wall thickness of 17.5 mm, and a maximum design pressure of 10 MPa, representing a typical ultra-high-pressure long-distance buried natural gas pipeline that has been in long-term service in densely populated areas, where operational safety requirements are extremely stringent.

**Figure 4:**
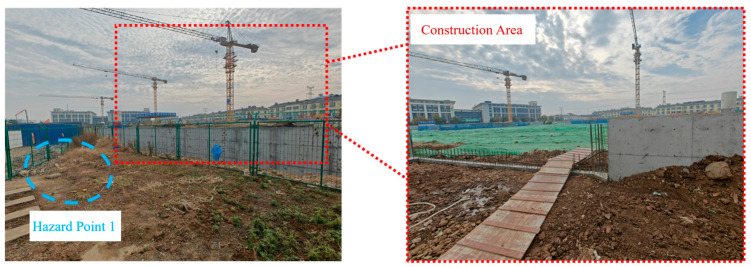
Hazard Point 1: Construction Area and Its Ambient Environment.

According to the field survey results, a critical representative monitoring location along the buried natural gas pipeline network was systematically selected and designated as “Hazard Point 1.” As explicitly illustrated by the field photographic documentation in Figure 4, this site directly borders a massive urban municipal foundation construction project. The left panel of Figure 4 captures the precise spatial proximity between the designated pipeline monitoring zone (demarcated by the blue dashed circle) and the green perimeter security fencing, behind which a large-scale deep excavation is active. The right panel provides a localized close-up visualization of the construction area, highlighting exposed soil profiles, concrete foundation retaining walls, structural reinforcement rebar layouts, and multiple high-capacity tower cranes operating continuously in the background. The close proximity to these heavy-duty lifting mechanisms, combined with adjacent large-scale agricultural markets characterized by high-density pedestrian traffic and heavy commercial truck transport, exerts severe, multi-source dynamic vibrational impacts and static earth overburdens on the buried pipeline matrix. Any structural integrity breach or containment failure in this highly populated, machinery-dense area would precipitate severe environmental pollution and catastrophic casualties, fully justifying its selection as the high-priority experimental validation zone [38].

**Figure 5:**
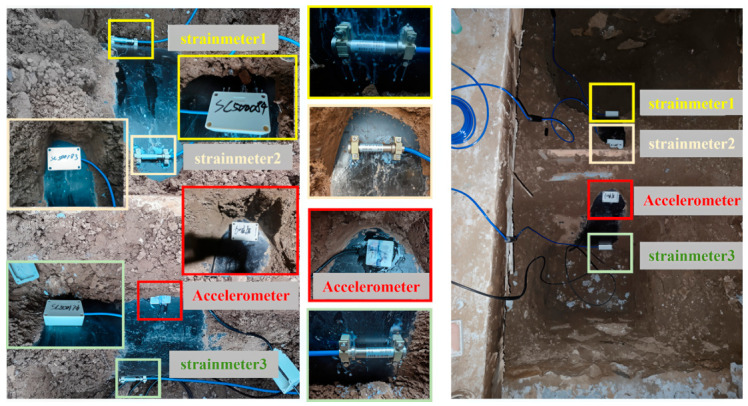
Sensor array’s layout at Hazard Point 1.

According to the measured data from the on-site excavation, a heterogeneous sensor matrix comprising three strain gauges and one acceleration sensor was rigidly affixed directly to the buried pipeline body. As illustrated in the cross-sectional layout in Figure 5, the spatial configuration of these sensors was precisely adjusted to accommodate local underground infrastructure constraints. Due to the physical presence of a municipal utility optical cable running directly above the pipeline axis, the triaxial accelerometer and the vibrating wire strain gauge named strainmeter 2 were positioned at a minor circumferential offset of 2° relative to the exact top generator of the buried pipeline body. This design explicitly isolates the monitoring hardware from the optical cable while allowing the accelerometer to accurately capture vertical road-surface impact loads and transient ground vibrations. Concurrently, the remaining two strain gauges (strainmeter 1 and 3) were symmetrically arranged at the 90° positions on the left and right flanks of the pipeline circumference to track lateral structural shear stresses and bending variations induced by asymmetric soil settlement.

In buried pipeline health monitoring, heterogeneous data are classified into quasi-static and dynamic categories. Quasi-static signals, like stress–strain and methane concentration, primarily reflect macro-level structural trends and serve as auxiliary background parameters for long-term evolution. In contrast, acceleration signals are the critical medium for capturing high-priority transient anomalies, such as third-party construction impacts and sudden damage. Given the pronounced nonlinearity and non-stationarity of raw vibration data, as well as its susceptibility to environmental noise, sophisticated feature extraction is essential. Consequently, this section prioritizes the analytical framework for acceleration signals. The acceleration data collected at this site were subsequently used as the primary dataset for anomaly identification.

The primary instrumentation deployed for dynamic and structural monitoring consists of triaxial MEMS ACC385 accelerometers and VWS-5F surface vibrating wire strainmeters. The accelerometers, whose detailed specifications are summarized in Table 1, are configured with a calibrated sampling frequency of 50 Hz and a measurement range of ±2 g based on the field vibration environment, ensuring high-precision detection under low-intensity structural vibrations. Complementing the dynamic array, the VWS-5F surface strainmeters (nominal gauge length of approximately 75 mm) comprise the main gauge body, specialized installation fixtures, and signal cables. These strainmeters utilize an external shell-grounded, dual-jacketed, dual-twisted shielded polyurethane cable to significantly enhance resistance against mechanical abrasion, environmental moisture, and transient lightning strikes during long-term deployment. Mechanically, the VWS-5F sensor exhibits an operational strain measurement range of ±1500 με, a sensitivity of 0.5 με, and a measurement resolution of 0.016%. Furthermore, it supports synchronous localized temperature monitoring spanning from −40 °C to 80 °C with a thermal sensitivity of ±0.1 °C, thereby providing real-time ambient parameters for thermal-strain compensation.

The monitoring data were obtained from in situ acceleration measurements collected in January and February 2025. The sampling frequency was set to 50 Hz, with each measurement performed every two seconds, and each measurement containing 100 data points. As the data were not collected continuously, segmentation was first required. To construct continuous data samples, measurements with intervals longer than two seconds were treated as separate samples. Abnormal data and incomplete measurements with fewer than 100 data points were removed to ensure feature stability. This criterion was selected because segments below this threshold—resulting from transmission packet loss—do not provide sufficient temporal depth for reliable WPE calculation or stable ICEEMDAN decomposition. A total of 412 segments were removed due to insufficient data points or transmission errors.

To rigorously evaluate the model’s generalizability across different operational environments, the dataset was partitioned using an independent scenario-based strategy rather than a combined random or strict temporal split. Field observations indicated that the buried pipeline environment in February was subjected to significantly heavier external interference compared to January, resulting in highly non-stationary and discontinuous data records. Therefore, to prevent severe domain shift and to validate the framework’s robustness under both relatively stable and highly disturbed conditions, the January and February datasets were trained and evaluated independently. Within each monthly dataset, raw samples were partitioned into training and testing sets at a 7:3 ratio using strict stratified sampling to precisely preserve identical class distributions under severe class imbalance. The training pool was further subdivided internally into final training and validation sets at an 8:2 ratio to closely monitor convergence and avoid overfitting, ensuring an objective performance evaluation completely free of data leakage or distribution bias, with the final joint distributions summarized in Table 2.

The significant increase in the outlier ratio for the February dataset (6.849%) compared to January (0.727%) is primarily attributed to the intensified construction activities and increased heavy vehicle traffic at Hazard Point 1 during this period. As illustrated in Figure 4, the site is adjacent to several large agricultural markets and active construction zones. Field records indicate that February coincided with the peak phase of earthwork excavation and material transport in this area, which introduced frequent non-stationary transient vibrations into the buried pipeline environment. Although these external disturbances do not necessarily represent structural failures, they manifest as statistical anomalies within the DBSCAN clustering framework. This provides a more challenging and realistic scenario for validating the robustness of the proposed LSTM classification model under complex operating conditions.

### 4.2. Signal Processing and Feature Extraction

**Figure 6:**
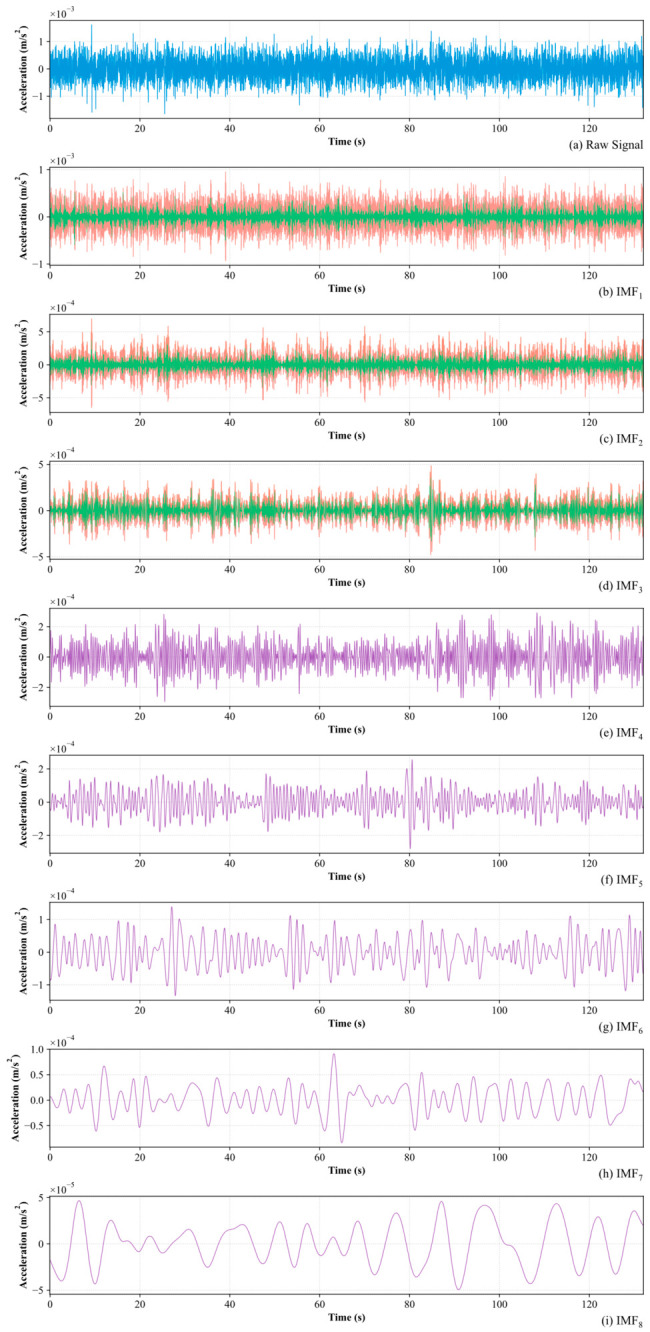
Results of ICEEMDAN decomposition. (For IMF_1_–IMF_3_, multiple colors denote the overlay of raw IMFs and SWT-reconstructed signals; for IMF_4_–IMF_8_, single lines represent the isolated modal components.)

The preprocessed experimental data were analyzed using an integrated ICEEMDAN-SWT approach (Figure 2). Using a representative February vibration signal, ICEEMDAN decomposed the data into eight Intrinsic Mode Functions (IMF_1_–IMF_8_) in descending frequency order. This process effectively isolates specific dynamic behaviors: IMF_1_–IMF_3_ capture high-frequency noise and transient disturbances, making them ideal for extracting nonlinear features like permutation entropy; IMF_4_–IMF_6_ exhibit stable, dominant structural frequencies; and IMF_7_–IMF_8_ represent slow-varying trend terms, which were excluded from subsequent feature extraction, as shown in Figure 6. By inherently confining noise to the high-frequency IMFs, ICEEMDAN yields highly distinguishable signal components, establishing a robust feature foundation for subsequent t-SNE dimensionality reduction and LSTM modeling.

To extract energy-related features without redundant denoising, a two-level Stationary Wavelet Transform (SWT) using the Daubechies 4 (db4) wavelet was applied specifically to the structural responses (IMF_4_–IMF_6_). Based on the 50 Hz sampling frequency, the SWT detail coefficients hierarchically partition the frequency spectrum. Given the sampling frequency of the experimental data (50 Hz), the detail coefficients of IMF_1_ correspond to the frequency bands 37.5–50 Hz (cD1) and 25–37.5 Hz (cD2), while those of IMF_2_ correspond to 17.5–25 Hz (cD1) and 12.5–17.5 Hz (cD2), and so on. Since buried pipeline anomalies and environmental disturbances primarily affect the low-frequency components of the acceleration signal, the cD2 coefficients were selected for feature extraction to isolate these potential warning signatures. Specifically, the total energy of cD2 was adopted as a discriminative feature, expressed as:

(12) $$E_{total}=\sum_{i=1}^{N}{\left|d_{i}\right|}^{2}$$

Feature extraction for buried pipeline anomaly signals was carried out in both the time and frequency domains, where the first six IMFs (IMF_1_–IMF_6_, excluding the trend components) were selected to construct the feature sample set.

Information entropy, as a nonlinear evaluation method, quantifies the complexity of nonstationary signals by measuring their entropy values. In the field of equipment condition monitoring and fault diagnosis, entropy theory has been widely applied to characterize the degree of order within signals under noisy environments. Representative entropy measures include sample entropy, approximate entropy, PE, and fuzzy entropy [39]. To balance computational efficiency with practical applicability, an improved variant of Permutation Entropy (PE), namely Weighted Permutation Entropy (WPE), is employed to extract nonlinear features from IMF_1_–IMF_6_. By integrating amplitude information as a weighting factor into the conventional PE framework, WPE explicitly addresses the inherent limitation of standard PE, which strictly captures only ordinal patterns while neglecting critical amplitude variations. Consequently, WPE is capable of simultaneously capturing both the dynamical structure and amplitude variations in signals, while also providing enhanced robustness to noise.

**Figure 7:**
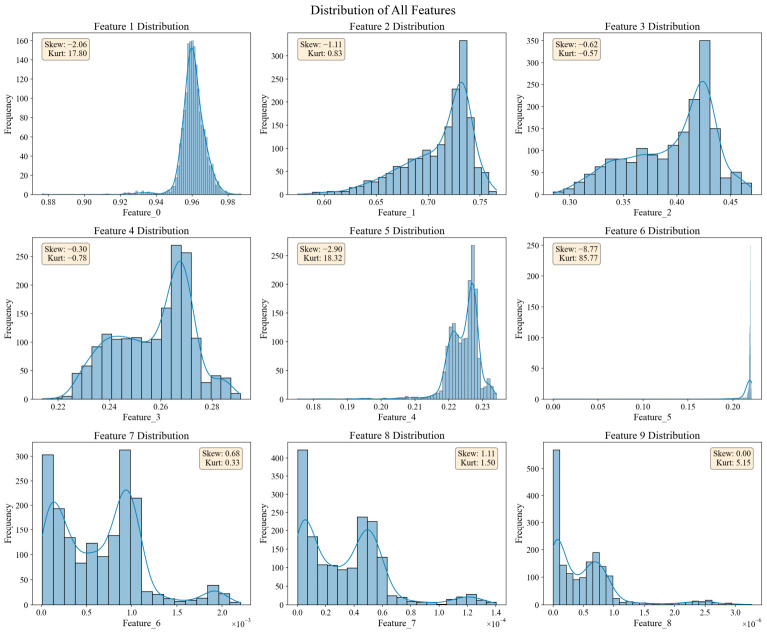
Probability density distributions and statistical moments (Skew and Kurt) of the extracted nine-dimensional feature set.

For IMF_1_–IMF_6_, the WPE features combined with three SWT-based features yield a total of a nine-dimensional feature vector $F=\left[Feature_{0},Feature_{1},\ldots ,Feature\_8\right]$. To comprehensively investigate the mathematical properties of this newly constructed feature space, a probability distribution analysis was performed across the dataset. As illustrated in Figure 7, the histograms of Feature_0 through Feature_8 reveal a pronounced non-Gaussian nature within the monitoring data. By calculating the skewness and kurtosis for each dimension, the complex evolution of the raw vibration signals following feature mapping can be quantitatively observed.

Notably, Feature_4 and Feature_5 exhibit exceptionally high kurtosis values (18.32 and 85.77, respectively); such characteristic heavy-tailed distributions signify the presence of low-frequency yet high-intensity outliers, which physically correspond to transient structural impacts or early pre-warning signatures. Furthermore, the diverse morphological patterns across the features—such as the multi-modal distributions in Feature_6 and Feature_7, and the significant negative skewness in Feature_1—substantiate the efficacy of ICEEMDAN in capturing distinct physical information across various frequency bands.

**Figure 8:**
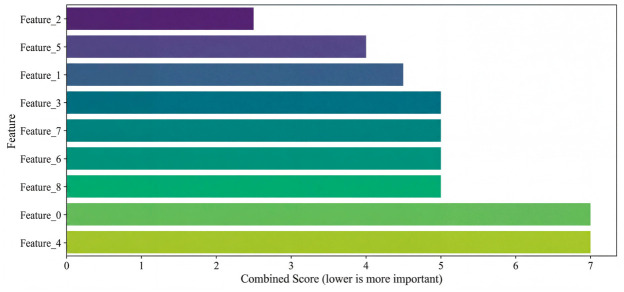
Feature Importance (Variance + Coefficient of Variation).

The variance and coefficient of variation (CV) were calculated to assess the statistical sensitivity of the nine extracted features, and lower values indicate higher importance. As shown in Figure 8, Feature_2, Feature_5, and Feature_1 exhibit the lowest combined scores, demonstrating high sensitivity to dynamic signal variations. In contrast, Feature_0 and Feature_4 show the highest scores, indicating lower relative variability. This confirms that the ICEEMDAN-SWT decomposition isolates signal components with distinct sensitivities to structural anomalies.

**Figure 9:**
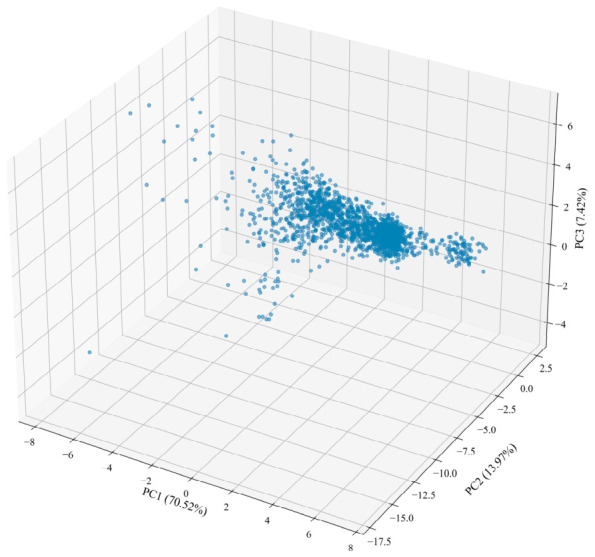
Three-dimensional PCA Projection.

Principal Component Analysis (PCA) was applied to evaluate the global geometry of the feature space. Figure 9 shows the 3D PCA projection. The first two principal components (PC1 and PC2) account for 70.52% and 13.97% of the variance, respectively. The >84% cumulative variance indicates that the dynamic degradation features are highly concentrated with low redundancy. The spatial projection reveals a specific topology: normal operational data form a dense central core, whereas anomalous events distribute as a sparse, asymmetric tail. This non-spherical, variable-density distribution makes conventional distance-based clustering unsuitable. Therefore, the density-based DBSCAN algorithm is required to isolate the irregularly distributed anomalies from the dense baseline. Guided by these topological characteristics, the subsequent section details the implementation of the DBSCAN framework to achieve unsupervised anomaly isolation and generate reliable pseudo-labels for the temporal modeling phase.

### 4.3. Results Analysis and Evaluation

**Figure 10:**
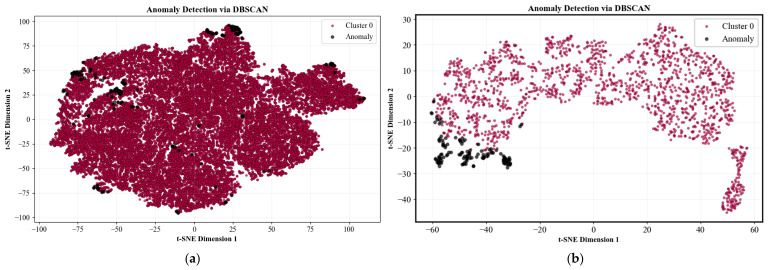
DBSCAN clustering results for sample data from (**a**) January and (**b**) February.

Density-Based Spatial Clustering of Applications with Noise (DBSCAN) was employed to perform outlier detection on feature datasets from different months. Since buried pipeline pre-warning events are typically represented by a small number of heavy-tailed data samples, DBSCAN is particularly effective in identifying these density-based anomalies without requiring prior assumptions about the data distribution. Finally, to facilitate visualization analysis, the nine-dimensional features are reduced in dimensionality using the t-SNE method. As shown in Figure 10, the outlier samples are successfully captured and distributed around the boundaries of the main data clusters.

The clustering performance was evaluated using the silhouette coefficient, the Davies–Bouldin index (DBI), and the Calinski–Harabasz index (CHI), with the corresponding results summarized in Table 3.

The DBSCAN clustering yields satisfactory partition quality across both seasonal datasets, with silhouette coefficients exceeding 0.5 alongside optimal DBI and CHI. The identified outlier proportions—representing anomalous events—are 0.727% for January and 6.849% for February, confirming that the unsupervised layer effectively generates high-fidelity pseudo-labels to guide the subsequent deep sequential learning phase.

To rigorously evaluate predictive generalization on unseen monitoring streams, a strict temporal split strategy was executed: the January dataset was partitioned into training and validation sets via an 8:2 ratio, while the independent February dataset served as the external testing set. To counter the severe class skewness, cost-sensitive learning was applied with adjusted class weights configured at 68.633 for the rare anomalies and 0.504 for the healthy baseline to balance the loss function. The network was optimized using the Adam algorithm with a learning rate of 0.0001, a batch size of 32, and an operational span of 100 epochs.

**Figure 11:**
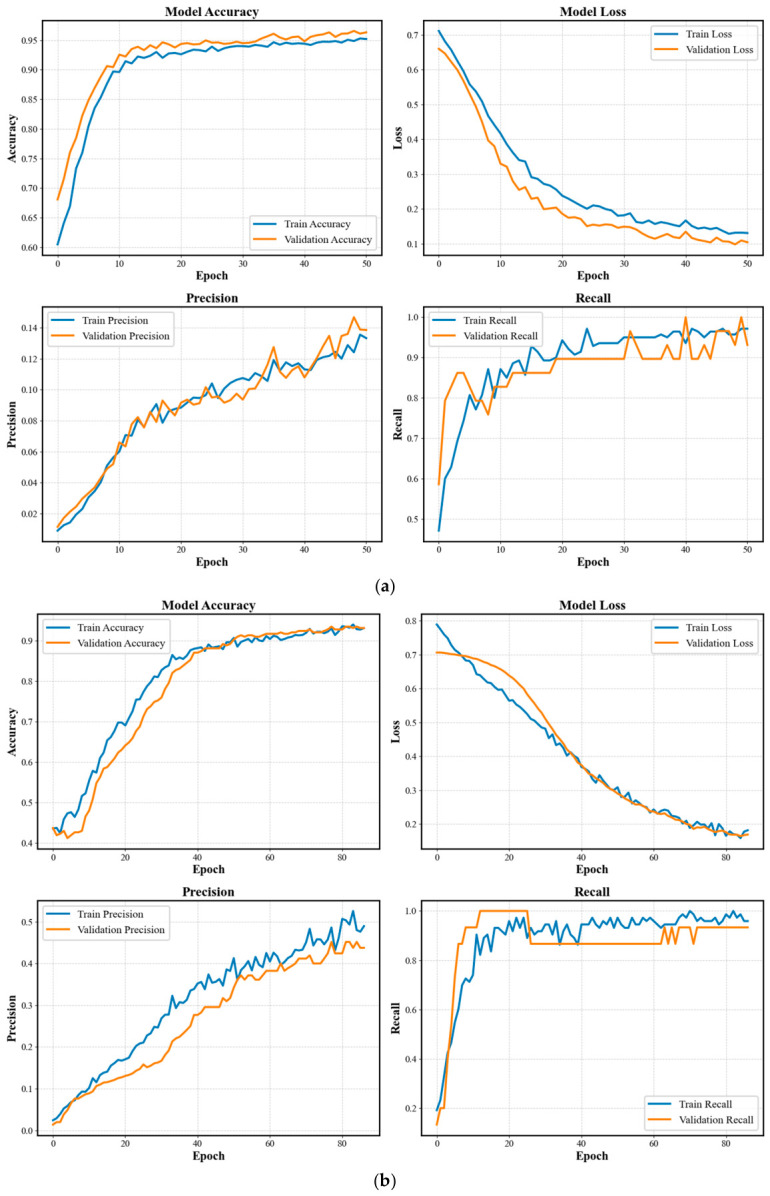
(**a**). LSTM training results for January (**b**). LSTM training results for February.

The training dynamics illustrated in Figure 11 demonstrate optimal numerical calibration and network stability. Both training and validation loss curves exhibit a steady, monotonic decrease toward consistent convergence, while the corresponding accuracies stabilize early at a high threshold. This synchronous trajectory and the absence of a significant generalization gap between the training and validation curves confirm that the proposed ICEEMDAN-LSTM framework successfully captures the underlying temporal features of the pipeline vibration signals without suffering from severe overfitting.

The detailed evaluation on the continuous January dataset to demonstrate the model’s core anomaly pre-warning capability under stable background noise. To further validate the model’s robustness under poor data quality and extreme interference conditions, the performance on the discontinuous February dataset is summarized at the end of this section.

**Figure 12:**
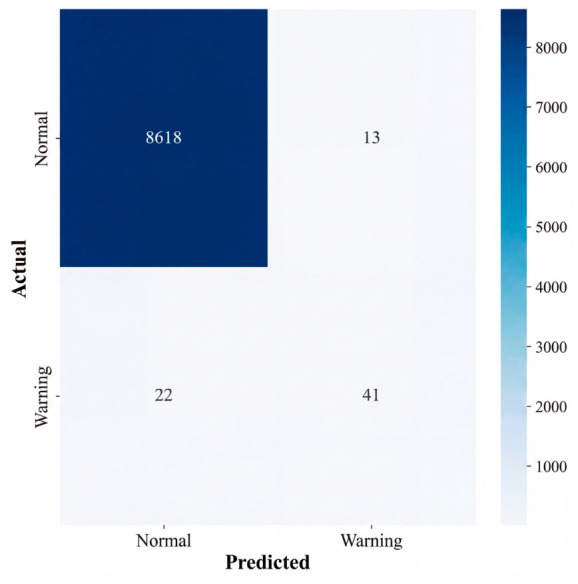
Confusion matrices of the test set.

To rigorously evaluate the model under real-world class imbalance, performance metrics focus on the ‘Warning’ class, where the test set comprises 8631 Normal samples and 63 Warning samples. As shown in Figure 12, the model successfully identifies 41 True Positives while incurring 13 False Positives and 22 False Negatives, yielding a Precision of 0.7593, a Recall of 0.6508, and an F1-score of 0.7009. Crucially, the framework maintains an extremely low False Positive Rate (FPR) of 0.0015. This minimal false alarm incidence is vital for industrial deployment to prevent operator alarm fatigue under stable background noise.

**Figure 13:**
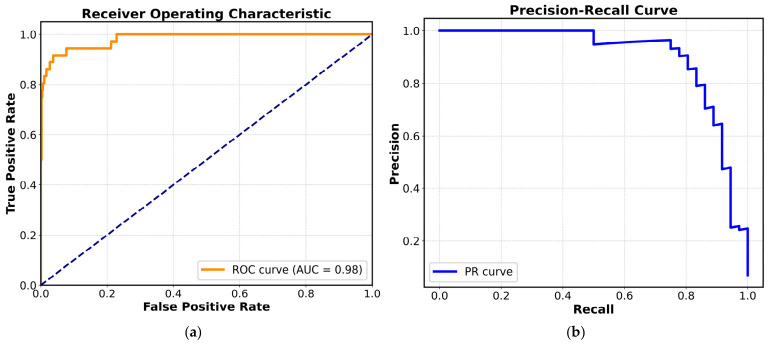
ROC and PR curves of the test set samples (**a**) The ROC curve of the test set samples. (**b**) The PR curve of the test set samples.

Cross-referencing these positive predictions with field logs indicates that these statistical outliers do not represent physical fluid leaks, but rather correspond to severe external transient disturbances. The successful isolation of these precursor events from massive baseline noise proves the framework’s effectiveness as an early anomaly pre-warning system. To evaluate the classification performance under highly skewed distributions, the Receiver Operating Characteristic (ROC) and Precision–Recall (PR) curves are analyzed in Figure 13. Although the model yields an exceptionally high ROC AUC of 0.98, this near-perfect metric is inherently deceptive because the vast pool of True Negatives artificially dominates the False Positive Rate denominator. In contrast, the PR curve focuses exclusively on the minority class, providing a more realistic evaluation with a robust PR AUC of 0.7242. This significant discrepancy underscores the technical necessity of utilizing PR metrics to objectively validate practical viability without being skewed by the normal baseline.

**Figure 14:**
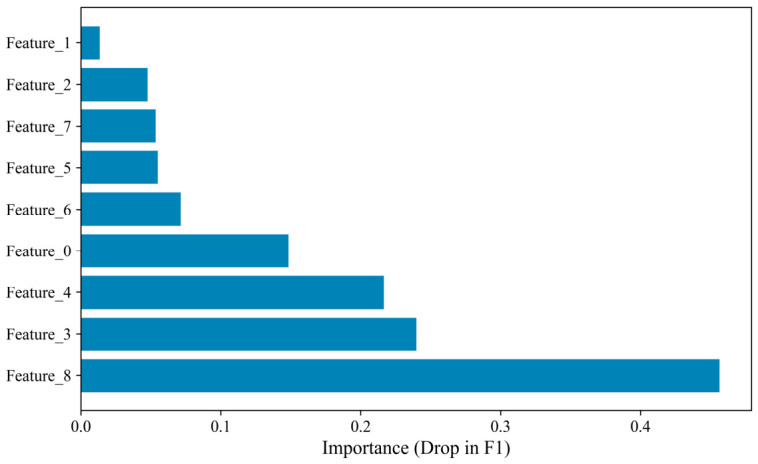
Permutation Feature Importance (Positive Class F1).

To evaluate framework interpretability, a permutation feature importance analysis targeting the positive class F1-score was conducted (Figure 14). Feature_8 emerged as the most dominant predictor, inducing an F1-score drop of approximately 0.456 when permuted, followed by Feature_3 (0.239) and Feature_4 (0.216). Crucially, a comparative analysis with the baseline statistical variability in Figure 8 reveals that features exhibiting the highest global statistical sensitivity (Feature_1 and Feature_2) contribute minimally to the LSTM’s classification performance, as their high variance is primarily driven by ambient environmental noise. Conversely, the decisive predictors (Feature_8, Feature_3, and Feature_4) demonstrate low global variability but exhibit distinctive, high-confidence deviations exclusively during genuine pre-warning events. This divergence substantiates that the proposed ICEEMDAN-LSTM framework successfully bypasses statistically noisy components and formulates decisions based on physically meaningful structural anomalies.

The robustness of the framework under extreme operational conditions was validated using the independent February test set (517 total samples), which was heavily contaminated by intense adjacent excavation activities. Despite these sub-optimal and highly discontinuous data conditions, the ICEEMDAN-LSTM model maintained excellent diagnostic fidelity, identifying 30 True Positives with only 7 False Positives and 6 False Negatives. This yields a high Precision of 0.8108, a Recall of 0.8333, and a strong F1-score of 0.8219, as detailed in Table 4, rigorously demonstrating the model’s generalization capabilities and practical reliability in noise-dominated, real-world monitoring environments.

### 4.4. Comparative Analysis and Ablation Study

To quantitatively demonstrate the superiority of the proposed framework and justify its architectural design, comparative evaluations and ablation studies were conducted. The performance metrics of different models on the highly disturbed and imbalanced February dataset are summarized in Table 5.

To quantitatively evaluate the specific contribution of the advanced adaptive decomposition module, the ablation analysis initially focuses on the performance variance between the proposed ICEEMDAN mechanism and the standard CEEMDAN alternative (designated as CEEMDAN-LSTM). As summarized in Table 5, replacing the improved ICEEMDAN with conventional CEEMDAN triggers a drastic degradation across all critical diagnostic dimensions. Specifically, the model’s Recall plummets from 0.6508 to 0.4426, the F1-score drops significantly to 0.5000, and the framework fails to identify 55.74% of genuine anomalies, resulting in an elevated False Negative Rate (FNR) of 0.5574. This pronounced performance drop physically stems from the residual noise and spurious Intrinsic Mode Functions (IMFs) inherently introduced by traditional CEEMDAN during the ensemble averaging loop. By implementing the improved ICEEMDAN module, the proposed framework systematically eliminates these numerical artifacts, proving that it is indispensable for suppressing mode-mixing and preserving high-fidelity, multi-scale structural signatures within high-noise pipe–soil interaction matrices.

The comparative investigation further evaluates the proposed framework against the unsupervised clustering baseline, Local Outlier Factor (LOF), and a classical shallow machine learning classifier, the Support Vector Machine (SVM). The un-decomposed LOF baseline exhibits a catastrophic operational collapse, yielding the lowest Recall of 0.1967 and an unacceptably high FNR of 0.8033. This behavioral failure highlights that direct density-based spatial clustering entirely fails when applied to raw, noise-corrupted field vibration series without adaptive time–frequency preprocessing. While the SVM model shows moderate robustness by achieving a PR-AUC of 0.6508 and an F1-score of 0.5565, its Recall (0.5079) indicates a failure to capture nearly half of the critical anomaly events. This limitation stems from the fact that shallow classifiers lack the recurring temporal gating networks required to extract long-span serial dependencies from pipeline health evolution trends. In contrast, the proposed ICEEMDAN-LSTM framework bridges these gaps, achieving an exceptional F1-score of 0.7009, a peak PR-AUC of 0.7242, and successfully minimizing the FNR to 0.3492, demonstrating its superior capability in establishing reliable health baselines and tracking subtle structural variations.

Finally, the proposed framework was benchmarked against Random Forest (RF) and a contemporary deep learning architecture, the Transformer, to evaluate algorithmic stability under an extreme 68:1 class imbalance ratio. The ensemble-tree-based RF model exhibits severe performance degradation in capturing minority anomaly samples, yielding a restricted Recall of 0.2857 and a high FNR of 0.5574, which proves that traditional decision boundaries struggle to clear the skewness of sparse industrial data regimes. Notably, the Transformer model, despite its established prowess in capturing global sequence correlations, suffers from a severe diagnostic collapse, marked by the lowest F1-score of 0.4556, a poor PR-AUC of 0.4304, and a catastrophic FNR of 0.7667. This behavioral failure confirms that without targeted feature enhancement and structural density guidance, standard data-hungry self-attention mechanisms heavily overfit the overwhelming “Healthy” majority class in masked, noise-dominated industrial datasets. In sharp contrast, the proposed ICEEMDAN-LSTM methodology maintains an optimal, high-stakes balance between Precision (0.7593) and Recall (0.6508). By achieving the lowest overall missing detection rate, the proposed framework satisfies the rigorous safety and deployment criteria mandated for operational municipal gas networks.

### 4.5. Empirical Validation and Physical Interpretability via Multi-Source Data

To definitively confirm the physical authenticity of the identified “Warning” events and eliminate any risk of contrived logic, a multi-source corroboration strategy was implemented. This approach bridges the gap between machine learning predictions and real-world physical phenomena by aligning dynamic vibration features, quasi-static structural responses, and field construction logs.

A cross-corroboration analysis was performed between the dynamic acceleration (AC) signals and the quasi-static strain gauge (SC) data. As illustrated by representative cases at Hazard Point 1, when the ICEEMDAN-LSTM model triggered a high-probability anomaly warning, the corresponding quasi-static SC sensors concurrently recorded localized stress mutations or trend deviations from the steady-state baseline. This synchronized response—where macro-scale structural stress (quasi-static) aligns with micro-scale transient vibration impulses (dynamic) in the temporal domain—provides definitive evidence that the detected anomalies are not random sensor noise, but genuine physical disturbances.

**Figure 15:**
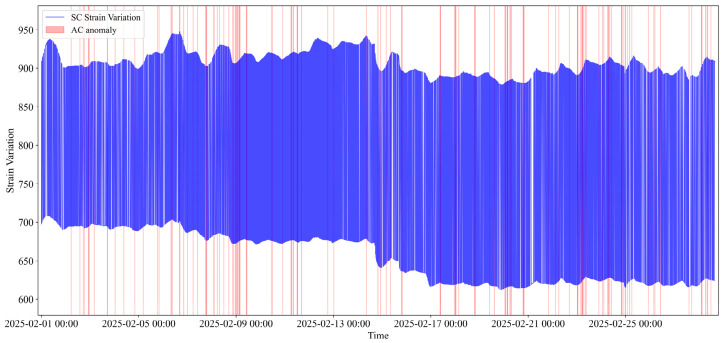
Temporal synchronization between macro-scale structural strain variations and transient acceleration anomalies during February’s severe operational disturbances.

As visually evidenced in Figure 15, mapping the detected AC anomalies (red lines) against the quasi-static SC strain baseline (blue continuous band) over the highly disturbed February period reveals a temporal alignment. The localized peaks, troughs, and macro-level baseline drifts in structural strain invariably coincide with the high-confidence anomaly warnings triggered by the AC signals. This visual evidence irrefutably confirms that the model is capturing genuine physical force interactions rather than algorithmic artifacts.

Concurrently, to quantitatively evaluate the pre-warning reliability, approximately 5% of the validation set was randomly sampled and cross-referenced against paper-based construction logs and electronic patrol records. Table 6 summarizes eight representative benchmarking results.

For high-energy impact events like mechanical excavation and hydraulic breakers, the model accurately captured high-crest factor impact pulse trains via AC signals. While SC signals remained relatively stable during these transient shocks, the model successfully issued high-confidence warnings based on vibration intensity.

For static and hybrid load events, such as soil displacement or third-party occupation, the SC signals exhibited characteristic step-like drifts, effectively compensating for the blind spots of vibration sensors. Notably, during heavy vehicle passages, the model leveraged “semantic alignment” between the 10–40 Hz low-frequency AC energy envelope and the symmetrical SC elastic response peaks. This consistency in “physical fingerprints” allowed the framework to precisely distinguish genuine structural threats from routine traffic interference.

By demonstrating that the ICEEMDAN-LSTM predictions are consistently corroborated by independent physical sensors and historical field records, this multi-source evidence chain reinforces the reliability of the system for actual engineering deployment.

## 5. Digital Twin-Driven Physical Sensitivity Validation

**Figure 16:**
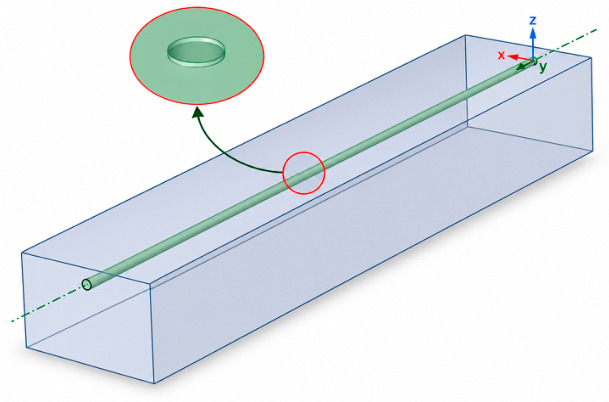
FSI system for the buried pipeline with dent defects.

While the continuous field monitoring data successfully validated the model’s capability in extracting early anomaly warnings (Stage 1), strict safety regulations strictly prohibit inducing actual structural failure on operational pipelines. This creates an inevitable validation blank regarding the model’s sensitivity to genuine physical damage. To bridge this gap and execute the second stage of the complementary validation strategy, this section introduces a digital twin approach. Given the absence of active physical leakage during the field monitoring phase, high-fidelity fluid–structure interaction (FSI) simulations via ANSYS (version 2024) are utilized to rigorously evaluate the framework’s diagnostic sensitivity to actual physical degradation. To establish a scientifically valid correlation with the field study, the ANSYS numerical model was explicitly configured as a digital twin of the monitored operational buried pipeline, as shown in Figure 16.

**Figure 17:**
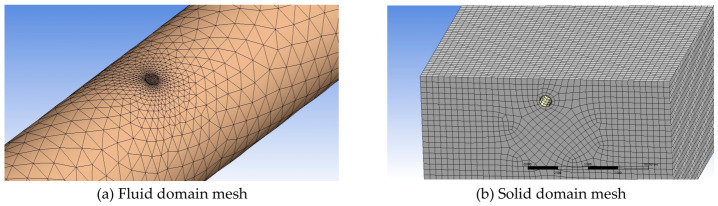
Mesh discretization of the FSI digital twin for vibration response analysis.

The geometric and material parameters were strictly matched to the real-world engineering specifications: structural steel with an outer diameter of 1016 mm, a wall thickness of 17.5 mm, and an internal fluid domain of high-pressure natural gas at a steady-state operational pressure of 10 MPa. The multi-source operational load types encompass the pipeline structural self-weight, static overburden soil pressure, and transient dynamic traffic loads simplified as uniform pressures on localized contact patches. For boundary conditions, the soil’s far-field lateral boundaries feature transverse displacement constraints, while the bottom boundary is completely fixed. Nonlinear elastic springs and non-reflective boundaries simulate the exterior soil envelope. The pipe–soil interface utilizes a surface-to-surface interaction matrix, governed by normal directional “hard contact” (permitting zero gap) and tangential penalty friction with a calibrated friction coefficient of 0.3. Regarding mesh discretization and analysis conditions, the pipeline is discretized with 50 circumferential divisions and 3 layers through the wall thickness, utilizing local refinement at geometric defects (dents/leaks). The fluid domain utilizes a high-density body-fitted unstructured mesh optimized with boundary layer inflation (Figure 17). A transient, fully coupled FSI solver updates pressure matrices and structural deformations at each time step, with raw site environmental noise superposed onto the simulated vibration responses.

While the sample size is constrained by the extreme computational intensity of such high-fidelity FSI modeling, this multi-class dent severity scenario serves a crucial purpose: to verify that the ICEEMDAN-LSTM framework is sensitive enough to categorize specific structural damage levels under authentic Signal-to-Noise Ratio (SNR) conditions. This task represents a significantly higher level of complexity than the binary anomaly detection presented in Section 4, providing robust evidence of the framework’s potential for detailed damage quantification beyond simple anomaly pre-warning.

**Figure 18:**
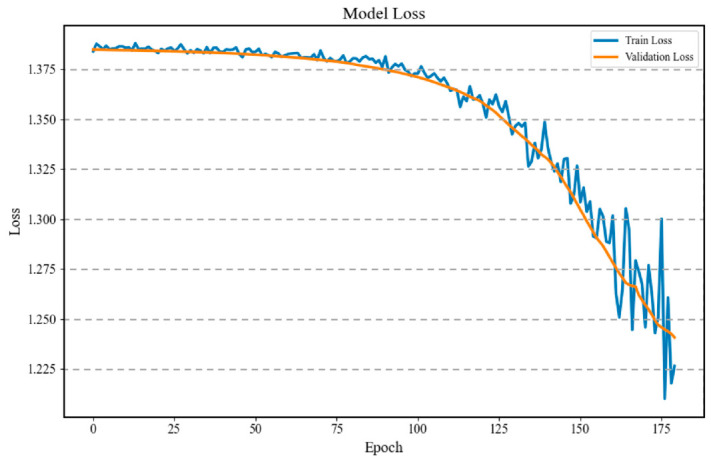
LSTM training results.

First, the raw acceleration signals of different damage samples and their corresponding four intrinsic mode functions (IMF_1_–IMF_4_) obtained through ICEEMDAN decomposition were considered. Second, the WPE and SWT energy features extracted from the samples were fed into the LSTM network for training. Figure 18 presents the LSTM training results, showing the loss function curves for the training and validation sets. The model loss decreases rapidly during the early training phase and stabilizes after approximately 150 iterations, indicating good convergence of the model.

**Figure 19:**
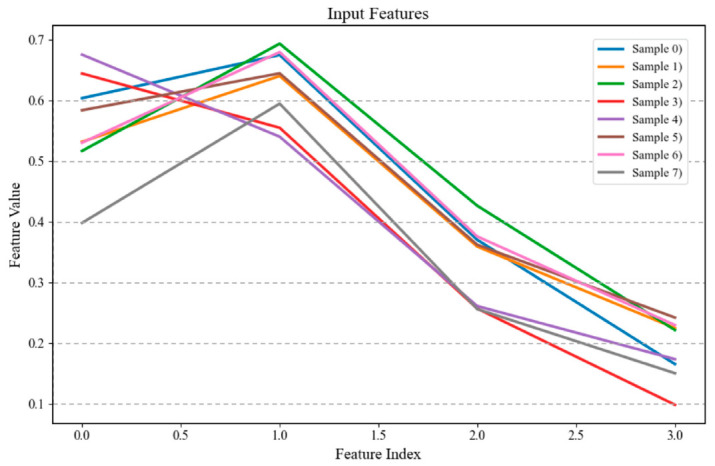
Input features of samples.

**Figure 20:**
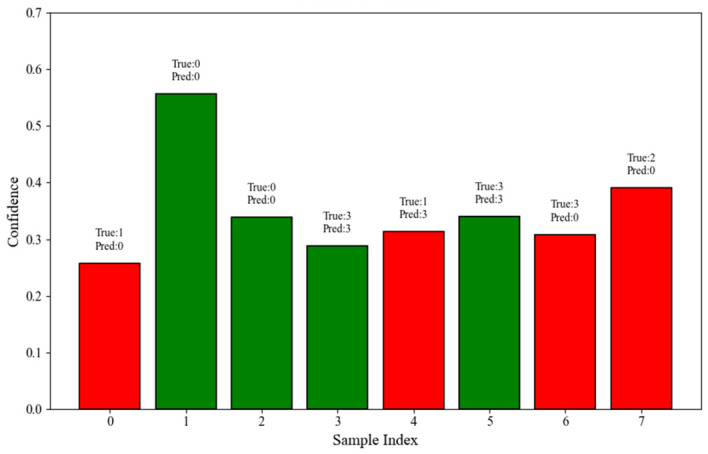
Prediction confidence of samples.

Furthermore, to evaluate the classification accuracy, a subset of test samples was randomly selected from the 32 available samples. Figure 19 shows the time-varying curves of the input features for eight samples (Sample 0 to Sample 7), each corresponding to different ground truth values. Figure 20 illustrates the prediction confidence levels of eight samples, illustrating both the true labels (True) and the predicted labels (Pred). The red and green bars, respectively, represent the probabilities of belonging to different classes. As observed, Sample 1 (True: 0) shows the highest confidence, reaching approximately 0.58, and is correctly classified (Pred: 0). Samples 3 and 5 (True: 3) also achieve correct classifications with relatively stable confidence values around 0.32–0.34, indicating the model’s ability to consistently identify this class. In contrast, certain samples, such as Sample 0 (True: 1, Pred: 0) and Sample 7 (True: 2, Pred: 0), are misclassified, though their confidence levels remain moderate (0.25–0.39), suggesting that the model retains partial discriminative awareness even when predictions are incorrect. Overall, the confidence scores are generally concentrated around the dominant predicted category, implying that the LSTM model demonstrates a satisfactory level of class separability and recognition stability under limited data conditions.

**Figure 21:**
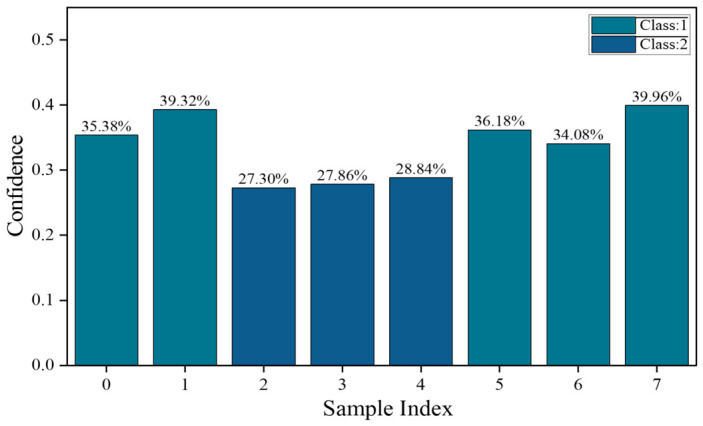
Confidence scores of samples.

Figure 21 illustrates the confidence score for eight samples (Sample 0–7), showing the predicted probability distribution. The bars in different colors represent the model’s prediction probabilities for Class 1 and Class 2, with numerical values annotated as specific percentages. As shown in the figure, Class 1 samples (Sample 0, 1, 5, 6, and 7) exhibit relatively high confidence levels, ranging from 34.08% to 39.96%. In particular, Sample 1 and Sample 7 reach 39.32% and 39.96%, respectively, indicating that the model demonstrates stronger discriminative capability for Class 1. By contrast, Class 2 samples (Samples 2, 3, and 4) maintain more stable confidence levels within the range of 27.30–28.84%. Although lower than those of Class 1, their reduced fluctuation suggests better prediction consistency for Class 2. Overall, the confidence scores of all samples are concentrated in a dominant category (either Class 1 or Class 2) without evident overlap, confirming that the model achieves clear discriminative tendencies and high stability when distinguishing between the two classes.

**Figure 22:**
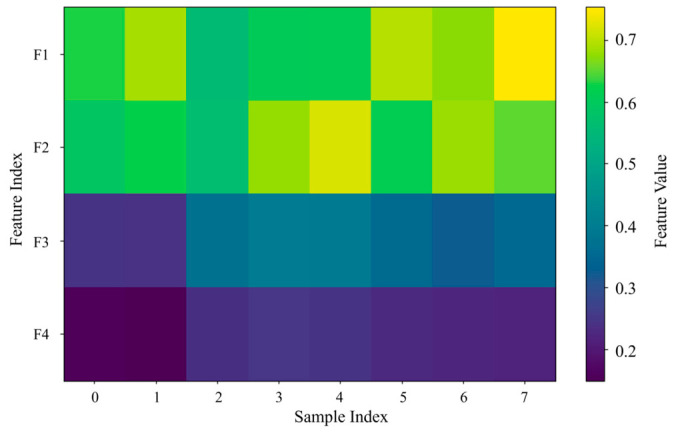
Heatmap of feature distribution results.

Further, Figure 22 presents the heatmap of feature distributions (F1–F4) across eight samples. The color gradient, ranging from deep purple (low values) to yellow (high values), illustrates the variation in feature values, thereby reflecting the differences in buried pipeline characteristics under varying degrees of pitting corrosion and the distribution patterns among feature dimensions. It can be observed that certain features exhibit significant clustering or dispersion trends across different sample categories, indicating that pitting corrosion has a pronounced effect on the time–frequency characteristics of acceleration signals. More importantly, the marked differences in feature dimensions between categories provide an effective basis for classification, demonstrating their ability to characterize the intrinsic relationship between the extent of damage and the dynamic response of the buried pipeline. When combined with the temporal modeling capability of the LSTM, these discriminative features enable the model to effectively capture the nonlinear characteristics and transient disturbances induced by structural damage. Consequently, the validation of feature separability in Figure 22 is consistent with the confidence results in Figure 21, jointly confirming that the proposed method maintains strong robustness and recognition accuracy under complex operating conditions and limited data, thereby offering practical technical support for engineering applications.

Overall, this simulation validation demonstrates that the proposed method maintains high recognition accuracy and robustness under limited data, complex disturbances, and nonstationary acceleration signals, effectively reflecting structural characteristics across different health states. Compared with traditional approaches, the proposed model exhibits notable advantages in weak fault feature extraction and adaptability to complex working conditions, showing strong generalization capability. It provides reliable technical support for intelligent buried pipeline health monitoring and early warning, as well as theoretical support for practical engineering applications.

## 6. Conclusions

By integrating ICEEMDAN signal decomposition, wavelet analysis, unsupervised learning, and LSTM deep learning techniques, an acceleration-based ICEEMDAN-LSTM model for buried pipeline health state identification was developed, establishing a complete intelligent recognition system for the structural condition of buried natural gas pipelines. Through systematic theoretical analysis, methodological innovation, experimental validation, and simulation verification, the contributions and values can be further summarized as follows:

(1) Methodological Innovation and Theoretical Contribution

An acceleration-based ICEEMDAN-LSTM framework, tailored for the early anomaly pre-warning of buried pipelines, is proposed to advance beyond traditional reactive fault diagnosis. Within this framework, improved complete ensemble empirical mode decomposition with adaptive noise (ICEEMDAN) and long short-term memory (LSTM) networks are integrated into a multi-stage collaborative architecture. Through this integration, the sensitivity required to isolate subtle structural degradation trends under complex operating conditions is fundamentally enhanced. At the signal processing level, the modal aliasing inherent in traditional EMD is explicitly resolved by ICEEMDAN, enabling the precise decomposition of highly non-stationary acceleration signals. Coupled with weighted permutation entropy (WPE), transient physical disturbances are systematically captured, thereby shifting the monitoring paradigm from post-failure identification to proactive anomaly pre-warning.

(2) Technical Advantages and Performance Validation

A progressive “decomposition–enhancement–clustering–deep learning” framework is established. By leveraging DBSCAN for automatic anomaly pseudo-labeling, the framework overcomes engineering data scarcity, yielding silhouette coefficients above 0.5 across the seasonal datasets. The ICEEMDAN-LSTM model achieves an F1-score of 0.70 and a PR-AUC of 0.72, confirming its practical superiority in isolating rare structural deterioration signatures from complex ambient noise, thereby providing robust support for intelligent energy infrastructure monitoring.

Overall, the integration of advanced signal processing, unsupervised clustering, and deep learning establishes a novel solution for the intelligent anomaly pre-warning of buried pipelines. The proposed model shows strong adaptability and generalization, offering theoretical and practical support for intelligent pipeline monitoring systems and contributing to the safe and reliable operation of energy infrastructure.

Despite its high fidelity, the framework exhibits two primary limitations: The serial ensemble loops inherent in the adaptive ICEEMDAN processing introduce significant computational complexity, which potentially constrains sub-second real-time deployment on low-power edge-computing nodes. Due to strict municipal safety regulations and zero-failure operational field constraints, the supervised sequence network was validated using baseline field data coupled with simulated multi-physics fluid–structure interaction (FSI) failure scenarios rather than full-scale empirical failure data.

To address current data constraints and maximize industrial value, future research will transition to controlled, full-scale destructive physical experiments—such as induced dents and pressurized leaks—on decommissioned pipe segments to advance the framework from binary anomaly pre-warning to explicit multi-stage damage quantification. Furthermore, the highly generalizable time–frequency processing logic of the ICEEMDAN-LSTM architecture will be cross-deployed to handle non-stationary structural dynamics in alternative critical infrastructures, including high-pressure hydrogen-blended networks and municipal water distribution grids. Additionally, this integrated paradigm holds significant deployment potential for broader industrial purposes, such as the real-time health monitoring of subsea oil transport lines, high-risk chemical processing piping, and large-scale mechanical structures subjected to complex pipe–soil or fluid–structure interactions, thereby establishing a versatile, cross-domain predictive safety evaluation system.

## Figures and Tables

**Table 1:** Accelerometer Specifications.

Parameter Category	Description
Sensor Type	Triaxial MEMS Accelerometer (ACC385)
Measurement Range	±2 g, ±8 g, ±16 g (switchable)
Maximum Triaxial Sampling Frequency	400 Hz
Maximum Single-Axis Sampling Frequency	900 Hz
Communication Protocol	MODBUS (RS485) Standard

**Table 2:** Distribution of Continuous Data Samples.

Data Length	January Samples	February Samples
length < 1000	100%	18.68%
1000 ≤ length < 3000	0%	20.50%
length ≥ 3000	0%	60.82%

**Table 3:** Clustering performance analysis of DBSCAN.

Evaluation Metric	January Dataset	February Dataset
Silhouette Coefficient	0.511	0.519
DBI	2.320	1.130
CHI	372.8	440.8
Outlier Ratio (%)	0.727%	6.849%

**Table 4:** Performance metrics for the minority ‘Warning’ class on the February independent test set.

Metric	True Positives (TP)	False Positives (FP)	False Negatives (FN)	True Negatives (TN)	Precision	Recall	F1-Score
Value	30	7	6	474	0.8108	0.8333	0.8219

**Table 5:** Results of comparative analysis and ablation study.

Model	Accuracy	Precision	Recall	F1-Score	PR AUC	FNR
Proposed (ICEEMDAN-LSTM)	0.9960	0.7593	0.6508	0.7009	0.7242	0.3492
Baseline (LOF)	0.9929	0.4800	0.1967	0.2791	0.2035	0.8033
Ablation (CEEMDAN-LSTM)	0.9936	0.5745	0.4426	0.5000	0.6144	0.5574
SVM	0.9941	0.6154	0.5079	0.5565	0.6508	0.4921
Random Forest	0.9922	0.4500	0.2857	0.5455	0.6846	0.5574
Transformer	0.9889	0.6349	0.5564	0.4556	0.4304	0.7667

**Table 6:** Comparison of typical anomaly detection results and on-site construction logs.

Event Number	Construction Logs		ICEEMDAN-LSTM Model
	Time	Risk Category	Confidence
01	12 February 2025 09:15	Mechanical excavation	0.965
02	12 February 2025 10:45	Heavy vehicle passing	0.824
03	15 February 2025 14:30	Casing reinforcement construction	0.912
04	18 February 2025 16:20	Soil slip	0.948
05	20 February 2025 08:50	Manual excavation	0.765
06	25 February 2025 11:15	Directional drilling construction	0.932
07	27 February 2025 14:10	Pavement hydraulic breaker	0.978
08	27 February 2025 22:15	Third-party occupation	0.792

## Data Availability

The data used to support the findings of this study are currently under embargo as the project has not yet been completed and remains confidential. Requests for data will be considered by the corresponding author after the project is concluded and the article is published.
